# Combination of LowDose Epigenetic Modifiers and TIC10 for the Activation of Antitumor Immunity and Inhibition of Tumor Growth in Gastrointestinal Cancer

**DOI:** 10.1002/cam4.71061

**Published:** 2025-07-17

**Authors:** Jianling Zou, Wentao Yang, Shuang Li, Fei Liu, Jinjia Chang, Wenhua Li, Mingzhu Huang, Xiaodong Zhu, Jianyin Zou, Weijian Guo, Zhiyu Chen

**Affiliations:** ^1^ Department of Medical Oncology Fudan University Shanghai Cancer Center Shanghai China; ^2^ Department of Oncology Shanghai Medical College, Fudan University Shanghai China; ^3^ Department of Colorectal Surgery Fudan University Shanghai Cancer Center Shanghai China; ^4^ Department of Gastrocolorectal Surgery General Surgery Center, The First Hospital of Jilin University Jilin China; ^5^ Department of Nuclear Medicine Fudan University Shanghai Cancer Center Shanghai China; ^6^ Department of Otolaryngology‐Head and Neck Surgery Otolaryngology Institute of Shanghai JiaoTong University, Shanghai Sixth People's Hospital Affiliated to Shanghai Jiao Tong University School of Medicine Shanghai China

**Keywords:** combination therapy, epigenetic modifiers, gastrointestinal cancer, TIC10, tumor microenvironment

## Abstract

**Background:**

Cytotoxic agents remain the mainstay treatment for advanced gastrointestinal cancer. However, the number of cytotoxic agents is limited, and the treatment effect is not satisfactory. Therefore, new agent and combination strategies are to be explored.

**Methods:**

The antitumor efficacy of low‐dose epigenetic modifiers (LD‐EMs) of 5‐azacytidine and entinostat, cytotoxic agents of paclitaxel, cisplatin, oxaliplatin, 5‐fluorouracil, and a novel cytotoxic agent TIC10, and the combination of LD‐EMs and cytotoxic agents was investigated in vivo. Flow cytometry and immunohistochemistry were conducted to analyze the immune phenotype in the tumor microenvironment. The proliferation and apoptosis analyses were performed in vitro.

**Results:**

LD‐EM therapy demonstrated superior tumor inhibition compared with commonly used chemotherapy in gastrointestinal cancer. A novel cytotoxic agent TIC10 resulted in weak to moderate tumor growth inhibition (TGI). LD‐EMs exhibited a more pronounced antitumor effect than TIC10 alone (CT26: TGI of 74.5% vs. 46.2%, respectively; *p* < 0.05; HNM007: TGI of 52.0% vs. 21.4%, respectively; *p* < 0.05; AKR: TGI of 53.8% vs. 10.1%, respectively; *p* < 0.05). The combination of TIC10 and LD‐EMs led to a more pronounced tumor reduction with tolerable toxicity. Analysis of the immune profiles showed increased percentages of CD45^+^ lymphocytes, CD3^+^ and CD8+ T cells, M1 macrophages, and dendritic cells, whereas decreased percentages of M2 macrophages and myeloid‐derived suppressor cells under treatment with LD‐EMs and combination therapy. Mechanistic studies revealed that LD‐EMs activated the RIG‐I–MAVS pathway, stimulated type I interferon responses, and subsequently promoted chemokine secretion. In contrast, TIC10 suppressed cell viability and induced cell apoptosis.

**Conclusions:**

LD‐EMs remodeled the tumor microenvironment to an immune‐promoting environment. Although TIC10 could suppress cell viability and induce cell apoptosis. A combination of LD‐EMs and TIC10 indicated a rational strategy through complementary mechanisms.

## 1 Introduction

Gastrointestinal (GI) cancer is among the most common types of cancer worldwide; it is characterized by high morbidity and mortality rates [1] and limited treatment options. Cytotoxic agents remain the mainstay treatment for advanced gastrointestinal cancer. However, the number of conventional cytotoxic agents is limited, and the effect of treatment is far from satisfactory. Remarkable advances have been made in the treatment of advanced GI cancer during the last few decades, including the introduction of targeted therapies and immune checkpoint inhibitors. However, the overall survival duration for patients with GI cancer is less than 3 years [2], making it important to explore new treatment options in these patients.

TIC10 is a small molecule imipridone that can upregulate endogenous tumor necrosis factor (TNF)‐related apoptosis‐inducing ligand (TRAIL) levels within tumor cells. It induced potent cytotoxic and cytostatic activities in various preclinical models, including colorectal cancer [3, 4]. In previous studies, TIC10 has exhibited exceptional safety, favorable pharmacokinetic and pharmacodynamic profiles, and promising efficacy in phase I/II trials [5, 6]. Apart from the cytotoxic effect, the role of TIC10 in the TME of GI cancer has not been investigated. The combination of TIC10 with other treatment modalities may also represent a new research direction.

Epigenetic modifiers (EMs) have shown promising therapeutic prospects in hematologic malignancies, and it has also reignited optimism about their potential application in the treatment of solid tumors. Historically, EMs have shown limited efficacy in solid tumors. However, in a phase I/II clinical trial, two patients with refractory advanced non‐small‐cell lung cancer achieved an objective and long‐lasting response with LD‐EM therapy [7]. In a select cohort of patients with advanced NSCLC whose disease had progressed following treatment with low‐dose 5‐azacitidine (5‐Aza) plus entinostat, robust and sustained tumor responses were observed upon subsequent exposure to immune checkpoint therapy [8], shedding light on the possible immunomodulatory properties of EMs [9]. This effect has been examined in various preclinical studies, revealing a promising therapeutic strategy in cancer treatment [10, 11, 12]. Importantly, our previous research showed that LD‐EMs can disrupt the trafficking of Myeloid‐derived suppressors (MDSCs), reshape the tumor microenvironment (TME), and impede metastasis in lung cancer, esophageal cancer, and breast cancer [13]. Previous studies have shown that reactivation of endogenous retroviruses (ERVs) can trigger a viral mimicry response, leading to the recognition of double‐stranded RNA by RIG‐I and MDA5, thereby activating type I interferon signaling and enhancing antitumor immunity [14, 15, 16, 17]. DNMT and HDAC inhibitors have been reported to enhance antitumor effects by reactivating silenced immune pathways [10, 18, 19, 20]. Moreover, epigenetic therapies—including DNMT and HDAC inhibitors—have been shown to induce ERV expression [21, 22]. Notably, the dual DNMT/HDAC inhibitor 15a has been demonstrated to activate the RIG‐I–MAVS pathway and promote chemokine secretion in breast cancer cells [23]. Building on these findings, we investigated whether low‐dose epigenetic modulators (LD‐EMs), either alone or in combination with TIC10, could induce viral mimicry and stimulate cytokine/chemokine production to enhance antitumor responses in GI cancer.

In this study, we investigated the efficacy of LD‐EMs and TIC10 in inhibiting tumor growth in GI cancer and assessed the potential synergistic effects of their combination. Additionally, we sought to elucidate how the combination of TIC10 and LD‐EMs impacts the TME.

## 2 Material and Methods

### 2.1 Drug Reagents and Dosing Schedule

TIC10 (Selleck Chemicals, Houston, TX, USA) was dissolved in dimethyl sulfoxide to a concentration of 50 mg/mL, 5‐Azacytidine (Sigma‐Aldrich. St. Louis, MO, USA) was dissolved in distilled water to a concentration of 5 mg/mL, and entinostat (Selleck Chemicals) was dissolved in dimethyl sulfoxide to a concentration of 1 mg/mL. These agents were aliquoted and stored at −80°C, with subsequent dilution to the required working concentrations before use. Paclitaxel was obtained from Haikou Pharmaceutical Factory (Hainan, China), and cisplatin for injection was obtained from Qilu Pharmaceutical (Shandong, China). Oxaliplatin and 5‐fluorouracil were obtained from Selleck Chemicals. All of them were diluted in saline to attain the necessary working concentrations prior to use.

### 2.2 Cell Lines and Animals

The murine esophageal squamous carcinoma cell lines HNM007 and AKR were generously provided by Prof. S. Singhal (University of Pennsylvania) and have been previously described [24, 25]. The murine colorectal carcinoma cell line CT26 was purchased from American Type Culture Collection (Manassas, VA, USA), and the MC38 cell line was purchased from the cell bank of the Chinese Academy of Sciences. Authentication and testing for Mycoplasma infection were conducted prior to the experiments. CT26 cells were cultured in Roswell Park Memorial Institute 1640 medium; all other cells were cultured in Dulbecco's modified Eagle's medium (Gibco, Billings, MT, USA). These media were supplemented with fetal bovine serum (Gibco) at a concentration of 10% v/v. Female C57BL/6 and Balb/c mice, aged 6–8 weeks, were obtained from Charles River Laboratories and maintained in a pathogen‐free environment throughout the study. All animal experiments were conducted in strict adherence to the animal experimental guidelines of the Institutional Animal Ethical Committee of Fudan University Cancer Hospital (FUSCC‐IACUC‐S20210481).

### 2.3 In Vivo Experiments

The mice received right flank subcutaneous injections of 1.0 × 10^5^ viable cells (HNM007, AKR, or CT26) suspended in 0.1 mL of phosphate‐buffered saline (PBS) and Matrigel (1:1, v/v). Drug administration commenced at 7–9 days after inoculation, once the tumor volume had reached approximately 100 mm^3^ (designated as day 1). The treatment regimen was as follows: 5‐Aza at a dose of 0.5 mg/kg, administered via daily subcutaneous injection; entinostat at a dose of 5.0 mg/kg, administered via daily intraperitoneal injection; chemotherapy, which comprised paclitaxel at a weekly dose of 20 mg/kg via intraperitoneal injection along with cisplatin at a biweekly dose of 3 mg/kg via intraperitoneal injection; Oxaliplatin at a weekly dose of 10 mg/kg via intraperitoneal injection along with 5‐fluorouracil at a weekly dose of 100 mg/kg via intraperitoneal injection; and TIC10, administered intragastrically at either 50 or 100 mg/kg twice per week. The tumor volume (mm^3^) was measured manually and calculated according to the formula V = 0.5 × L × W^2^, where L is the length (mm) and W is the width (mm). Tumor growth inhibition (TGI, %) was calculated using the formula TGI = (1−Δ*T*/Δ*C*) × 100%, where Δ*T* is the change in tumor volume in the drug‐treated group on the final day of the study and Δ*C* is the change in tumor volume in the control group on the final day of the study. Toxicity during the treatment period was evaluated based on a > 10% decrease in body weight.

### 2.4 Flow Cytometry Analysis

Flow cytometry was employed for immune profiling analysis after sample preparation. Tumor tissues were enzymatically digested for 30 min at 37°C using a dissociation kit (Miltenyi Biotec, Bergisch Gladbach, Germany), then minced and passed through a 70‐μm cell strainer to yield a single‐cell suspension. The cell count was determined, and the cells were incubated with rat monoclonal anti‐CD16/CD32 antibody (Fc block antibody) in PBS for 30 min at 4°C. Next, the cells were washed with PBS and stained with specific antibodies. For the identification of intracellular antigens, the cells were fixed and permeabilized using fixation/permeabilization buffer (BD Biosciences, Franklin Lakes, NJ, USA) for 30 min at 4°C, followed by washing and staining with intracellular antibodies for an additional 30 min at 4°C. All antibodies used in this analysis are described in detail in Table S1. Flow cytometry analyses were performed using a BD FACSCelesta Cell Analyzer (BD Biosciences).

Data analyses were conducted using ModFit v4.0 software. For cell apoptosis analysis, cells were exposed to different reagents for 48 h, followed by staining with annexin V–fluorescein isothiocyanate and 7‐aminoactinomycin (BD Biosciences) at room temperature in the dark for 15 min. Flow cytometry was performed using a BD FACSCalibur System (BD Biosciences), and analyses were completed within 30 min. Cell apoptotic data were analyzed using FlowJo v10.8.1 software (BD Biosciences).

### 2.5 Cell Counting Kit‐8 Assay

Viable CT26 cells were seeded into individual wells of 96‐well plates (2 × 10^3^ cells per well). The cells were subjected to treatment with specific concentrations of TIC10. Following a 48‐h incubation period, cell viability was assessed using a Cell Counting Kit‐8 assay (Dojindo Laboratories, Kumamoto, Japan) in accordance with the manufacturer's guidelines. The optical density at 450 nm was then measured using a microplate reader (Bio‐Rad Laboratories, Hercules, CA, USA).

### 2.6 CellTiter‐Lumi Assay

Viable CT26 cells were evenly distributed into individual wells of 96‐well plates at a density of 2 × 10^3^ cells per well, treated with specific concentrations of 5‐Aza and/or entinostat, and incubated for 48 h. Then, 100 μL of CellTiter‐Lumi luminescence detection reagent (Beyotime Biotechnology, Shanghai, China) was introduced into each well, followed by gentle shaking at room temperature for 2 min and incubation for 10 min. Chemiluminescence signals were detected using a microplate reader (SpectraMax iD3; Molecular Devices, San Jose, CA, USA).

### 2.7 Hematoxylin & Eosin (H & E) Staining, Immunohistochemistry (IHC), and Terminal Deoxynucleotidyl Transferase‐Mediated dUTP Nick‐End Labeling (TUNEL) Assays

Histopathologic examination (H&E) of CT26 tumors after different treatments was performed using an H & E staining kit (C0105; Beyotime Biotechnology), according to the standard method. The primary antibody of Ki‐67 and secondary antibodies were obtained from Servicebio Technology (Wuhan, China), and the antibody for CD3, CD4， CD8, PD‐1, PD‐L1, CTLA‐4 and LAG‐3 were from Huabio (Hangzhou, China). Mouse tumor samples were formalin‐fixed and embedded in paraffin, and 5‐μm‐thick sections were meticulously sliced and affixed onto slides. The sections underwent comprehensive processing and were subjected to IHC analysis. A TUNEL assay was conducted by Servicebio Technology. Ultimately, the acquisition of images was accomplished with a microscope manufactured by Olympus (Japan).

### 2.8 Western Blots

Tumor tissues from mice were lysed, and protein concentrations were quantified. Protein samples were diluted to a uniform concentration of 10 μg/μL and separated by 8%–12% SDS‐PAGE. Following electrophoresis, proteins were transferred onto nitrocellulose membranes (GE Healthcare, Piscataway, NJ). Membranes were incubated overnight at 4°C with the appropriate primary antibodies diluted in 5% BSA, followed by washing and incubation with secondary antibodies at room temperature for 1 h. The primary antibodies used in this study—TBK1, p‐TBK1 (S172), MDA5, RIG‐I, and GAPDH—were all obtained from Abclonal (China). Protein signals were detected using ECL Plus Western Blotting Detection Reagents (GE Healthcare).

### 2.9 RNA Extraction and Quantitative Real‐Time PCR

Total RNA was extracted from tumor tissues using the RNeasy Kit (Qiagen) and TRIzol reagent (Invitrogen, Carlsbad, CA, USA) according to the manufacturers' protocols. RNA samples with OD260/OD280 ratios between 1.9 and 2.0 were considered to be of good quality. Reverse transcription and quantitative PCR were performed to measure the expression levels of CCL5, CXCL9, CXCL10, and the internal control GAPDH. Relative gene expression was calculated using the comparative *C*_t_ (ΔΔ*C*_t_) method. The primer sequences used were as follows: *CCL5*: Forward 5′‐TTTGCCTACCTCTCCCTCG‐3′, Reverse 5′‐CGACTGCAAGATTGGAGCACT‐3′; *CXCL9*: Forward 5′‐GGAGTTCGAGGAACCCTAGTG‐3′, Reverse 5′‐GGGATTTGTAGTGGATCGTGC‐3′; *CXCL10*: Forward 5′‐CCAAGTGCTGCCGTCATTTTC‐3′, Reverse 5′‐GGCTCGCAGGGATGATTTCAA‐3′.

### 2.10 Statistical Analyses

Data analyses were conducted using GraphPad Prism v8.0 (GraphPad Software). All experiments were conducted using biological and technical triplicates. Error bars in graphs represent means ± standard deviation unless otherwise specified. For datasets conforming to a normal distribution, the two‐tailed Student's *t*‐test was applied for statistical evaluation, and statistical significance was evaluated at *p* < 0.05, *p* < 0.01, and *p* < 0.001.

## 3 Results

### 3.1 Inhibition of Tumor Growth by LD‐EMs in Subcutaneous Tumor Models

**FIGURE 1:**
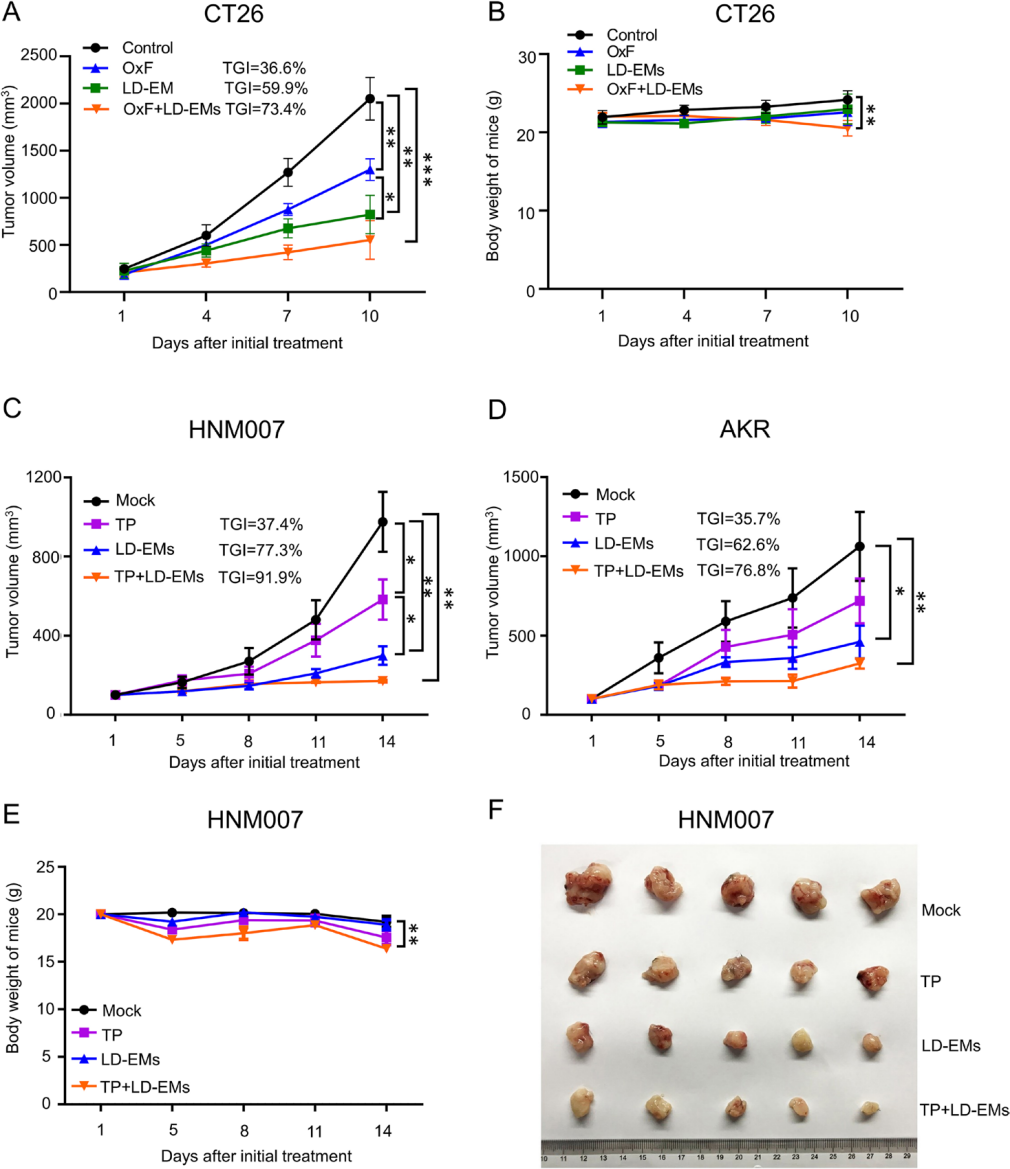
The antitumor effect of LD‐EMs on GI cancer models. The effect of chemotherapy, LD‐EMs, and chemotherapy plus LD‐EMs on tumor growth (A) and body weight (B) in the CT26 mouse model. 5‐fluorouracil (100 mg/kg) and oxaliplatin (10 mg/kg) once a week. The effect of chemotherapy, low‐dose epigenetic modifiers, and chemotherapy plus LD‐EMs on tumor growth in HNM007 (C) and AKR mice (D). (E) The effect of chemotherapy, LD‐EMs, and chemotherapy plus LD‐EMs on body weight in HNM007 mice. (F) Images of tumors after chemotherapy, LD‐EMs, and chemotherapy plus LD‐EMs in HNM007 mice at day 14. Mock: Vehicle. OxF: Oxaliplatin 10 mg/kg/week, intraperitoneal (ip); 5‐fluorouracil 100 mg/kg/week intraperitoneal (ip). TP: Paclitaxel 20 mg/kg/week, intraperitoneal (ip), cisplatin 3 mg/kg, ip, twice a week. LD‐EMs: 5‐Aza 0.5 mg/kg/day subcutaneous (sc), entinostat 5 mg/kg/day (ip). *N* = 5. All bars represent mean ± SD. *p < 0.05; **p < 0.01; ***p < 0.001.

In our prior study, epigenetic therapy was shown to impact lung metastasis by reshaping the pre‐metastatic niche [13]. To assess the in vivo effects of LD‐EMs on gastrointestinal cancer, colorectal cancer with the CT26 cell line and esophageal cancer mice models with the HNM007 and AKR cell lines were established subcutaneously. Four groups were divided: mock (vehicle), LD‐EMs (5‐Aza + entinostat), chemotherapy (oxaliplatin+5‐fluorouracil for CT26 mice model, paclitaxel + cisplatin for HNM007 and AKR mice models), and LD‐EMs + chemotherapy (Figure 1A–F). Comparative analysis with the mock group revealed significant suppression of tumor growth in the LD‐EM group, with TGI rates of 59.9%, 77.3%, and 62.6% in CT26, HNM007, and AKR mice, respectively (both *p* < 0.05) (Figure 1A,C,D). In CT26 and HNM007 mice, LD‐EM therapy demonstrated superior tumor inhibition compared with commonly used chemotherapy alone (TGI of 59.9% vs. 36.6% for CT26; 77.3% vs. 37.4% for HNM007, respectively; *p* < 0.05). Similar trends of tumor inhibition were observed in AKR mice, although statistical significance was not reached. Importantly, LD‐EMs were tolerated well and did not induce significant weight loss compared with the mock group (Figure 1B,E). However, the combination of LD‐EMs with traditional chemotherapy did not provide a superior effect compared to LD‐EMs alone but did lead to greater weight loss (Figure 1A–E), indicating the importance of exploring new treatment options and combination strategies. Figure 1F shows a representative image of tumors dissected from HNM007 mice on day 14.

### 3.2 Combined Efficacy of LD‐EMs and TIC10 In Vivo

**FIGURE 2:**
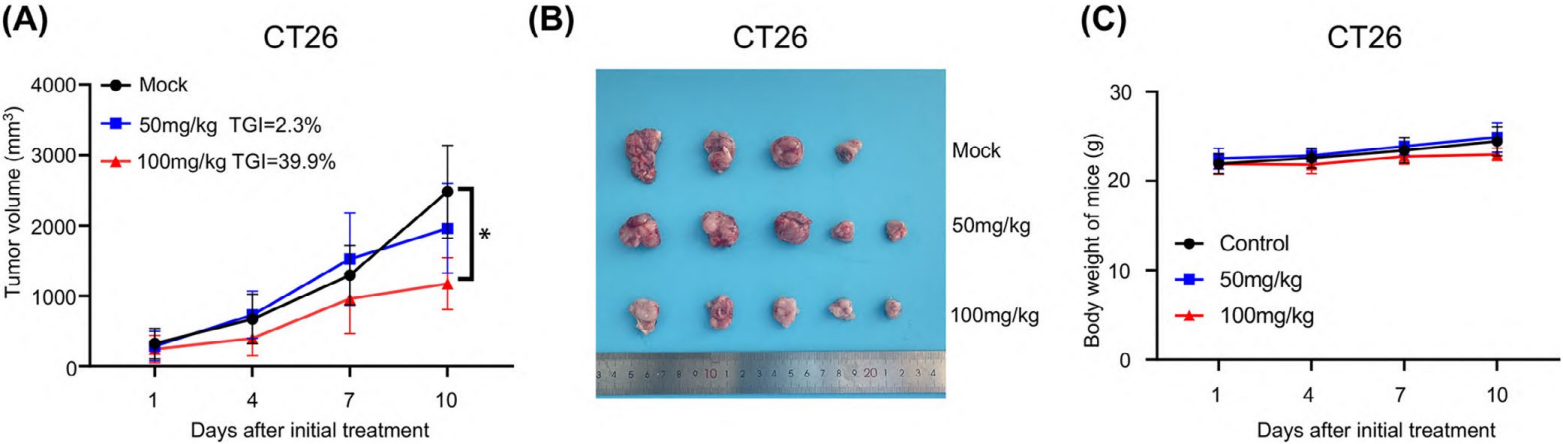
The antitumor effect of LD‐EMs on colorectal cancer models. (A) The effect of different dosages of TIC10 (50 mg/kg or 100 mg/kg) on tumor growth in CT26 mice. (B) Images of tumors after TIC10 treatment in CT26 mice at day 10. (C) The effect of TIC10 on body weight in HNM007 mice. TIC10 was administered via intragastric administration at either 50 mg/kg or 100 mg/kg, twice a week. *N* = 5. All bars represent mean ± SD. *p < 0.05.

We investigated the antitumor effects of varying doses of TIC10 in a CT26 model using BALB/c mice (Figure 2). TIC10 as a single agent moderately inhibited CT26 tumor growth at a dosage of 100 mg/kg twice weekly, resulting in a TGI of 39.9% (*p* = 0.04). However, at a dosage of 50 mg/kg twice weekly, TIC10 failed to impede tumor growth, yielding a TGI of only 2.3% (*p* > 0.05) (Figure 2A). Figure 2B shows a representative image of tumors harvested from CT26 mice on Day 10. Importantly, neither TIC10 dosage (50 mg/kg twice weekly or 100 mg/kg twice weekly) induced significant weight loss in the mice (Figure 2C).

**FIGURE 3:**
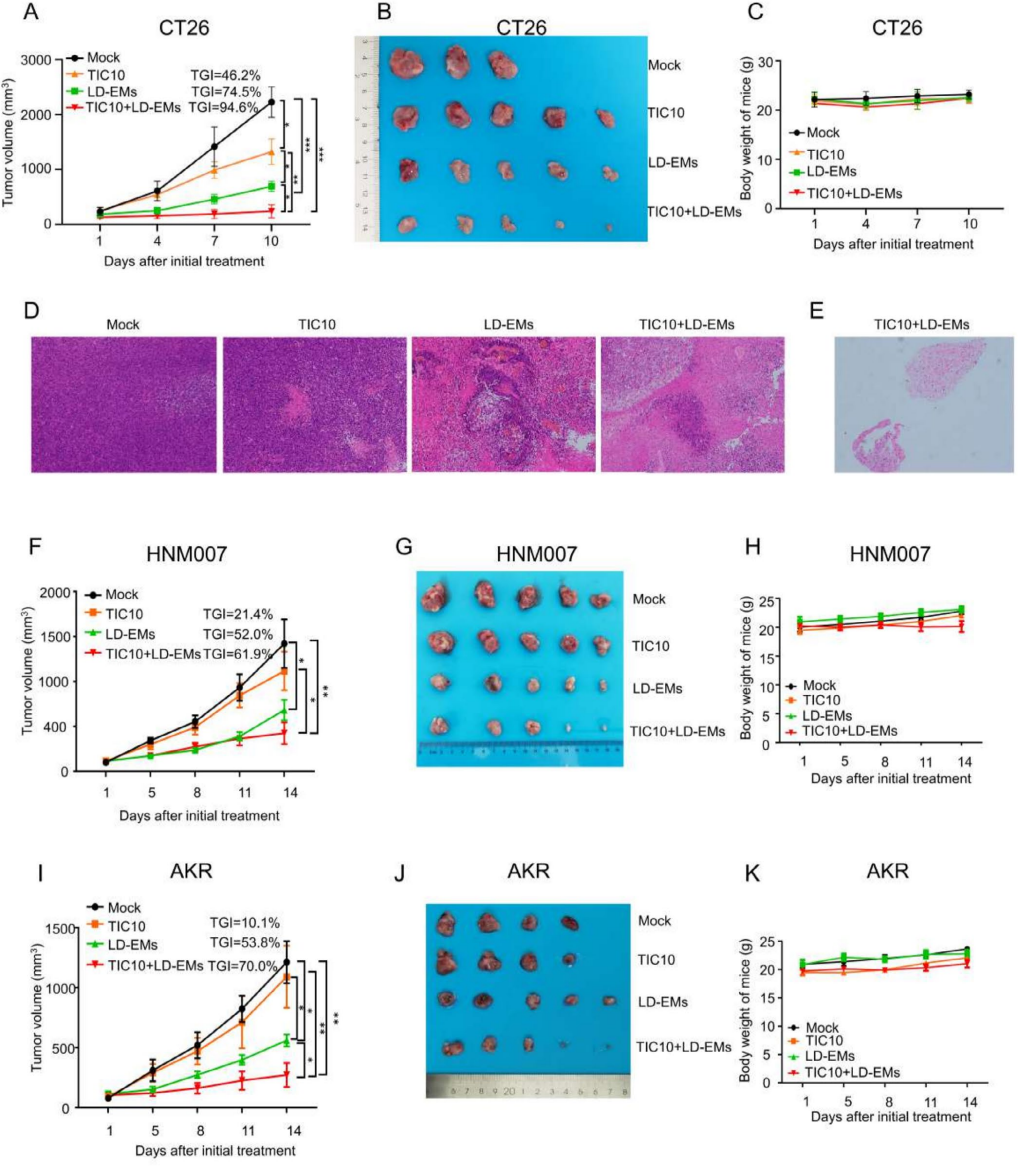
TIC10, LD‐EMs alone, or in combination, could decrease tumor growth in GI cancer models. (A) The effect of TIC10, LD‐EMs alone, or in combination on tumor growth in the CT26 mouse model. (B) Images of tumors after different treatments in the CT26 mouse model at day 10. (C) The effect of different treatments on body weight in the CT26 mouse model. (D) Representative images of tumor necrosis and lymphocyte infiltration in mice receiving different treatments. (E) A typical picture of one tumor exhibited complete regression after the combination treatment of LD‐EMs and TIC10, with extensive lymphocyte infiltration and no tumor cells within the tissue. (F) The effect of TIC10, LD‐EMs alone, or in combination on tumor growth in the HNM007 mouse model. (G) Images of tumors after different treatments in the HNM007 mouse model at day 14. (H) The effect of different treatments on body weight in the HNM007 mouse model. (I) The effect of TIC10, LD‐EMs alone, or in combination on tumor growth in the AKR mouse model. (J) Images of tumors after different treatments in the AKR mouse model at day 14. (K) The effect of different treatments on body weight in the AKR mouse model. Tumors from the CT26 mouse model were harvested on day 10 after the initial treatment, whereas those from the HNM007 and AKR models were collected on day 14. The slides were stained with H&E; images were captured at 100× magnification. TIC10 was given via intragastric administration at 100 mg/kg, administered twice a week. LD‐EMs: 5‐Aza 0.5 mg/kg/day subcutaneously (sc), entinostat 5 mg/kg/day intraperitoneally (ip). *N* = 5. All bars represent mean ± SD. *p < 0.05; **p < 0.01; ***p < 0.001.

Building upon our previous findings indicating the capacity of LD‐EMs to reshape the TME and inhibit tumor growth in GI cancer, we investigated the combined impact of LD‐EMs and TIC10 in GI cancer mouse models (Figure 3). In the CT26 mice model, LD‐EMs exhibited a more pronounced antitumor effect than TIC10 alone (TGI of 74.5% vs. 46.2%, respectively; *p* < 0.05). Notably, the combination of TIC10 and LD‐EMs led to a substantial reduction in tumor growth, with a TGI of 94.6% (*p* < 0.001) (Figure 3A,B). Furthermore, the combination of TIC10 and LD‐EMs was tolerated well and did not induce significant weight loss in the mice (Figure 3C). H & E staining to assess tumor status revealed increased necrosis and lymphocyte infiltration in tumors treated with LD‐EMs and combination therapy (Figure 3D). Additionally, one tumor exhibited complete regression, characterized by extensive lymphocyte infiltration within the tissue (Figure 3E). The antitumor efficacy of the combination of TIC10 and LD‐EMs was confirmed in a HNM007 and AKR mouse models of esophageal cancer (Figure 3F–K). TIC10 alone exhibited limited tumor growth inhibition, with a TGI of 21.4% in HNM007 and 10.1% in AKR, showing no significant effect (Figure 3F,G,I,J). However, the combination of TIC10 and LD‐EMs resulted in a marked reduction in tumor growth, achieving a TGI of 61.9% and 70.0%, respectively (*p* < 0.01) (Figure 3F,G,I,J), without inducing significant weight loss (Figure 3H,K).

### 3.3 Combination Therapy‐Induced Reprogramming of the TME to an Active Immune Phenotype

**FIGURE 4:**
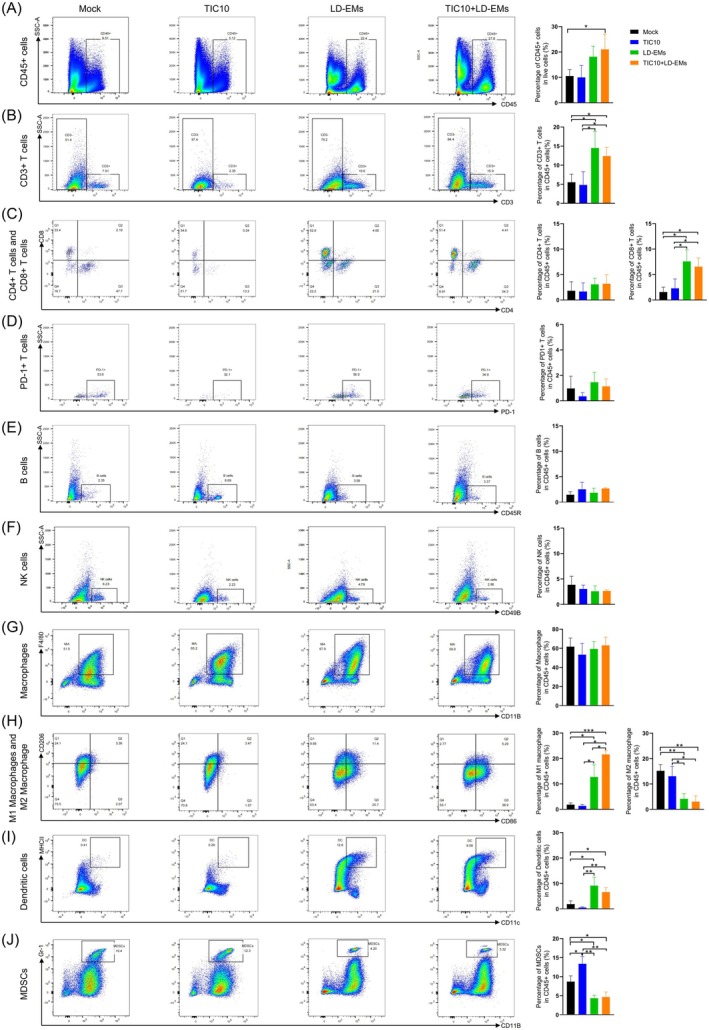
The immune profiles after treatment with TIC10, LD‐EMs, or combination therapy. Representative images of different immune profiles (left) and percentage (right) after treatment: (A) CD45+ cells; (B) CD3+ T cells; (C) CD4+ T cells and CD8+ T cells; (D) PD1+ T cells; (E) B cells; (F) NK cells; (G) Total macrophages; (H) M1 macrophages and M2 macrophages; (I) Dendritic cells; (J) MDSCs. (*n* = 3 mice per group). Data were analyzed by flow cytometry. *N* = 3. All bars represent mean ± SD. *p < 0.05; **p < 0.01; ***p < 0.001.

To further examine the mechanisms underlying the efficacy of the combination therapy, we performed flow cytometry analysis to scrutinize the immune cell composition within the TME in vivo. The analytical process is described in detail in Figure S1. The comparative analysis revealed that the percentage of CD45+ cells in the TME was significantly higher in the combination therapy group than in the mock group (21.03% ± 6.01% vs. 10.52% ± 2.52%, respectively; *p* < 0.05), whereas neither TIC10 (10.01% ± 4.70%) nor LD‐EMs (18.13% ± 4.15%) induced a significant increase in CD45+ cells (Figure 4A).

The percentage of CD3+ T cells was significantly higher in tumors treated with LD‐EMs (13.85% ± 5.34%) and combination therapy (10.40% ± 5.18%) than in the mock group (4.86% ± 2.68%, *p* < 0.05 for both), whereas TIC10 did not significantly increase the percentage of CD3+ T cells (4.83% ± 3.45%) (Figure 4B). In addition, the percentage of CD8+ T cells was significantly higher in tumors treated with LD‐EMs (7.59% ± 2.31%) and combination therapy (5.89% ± 2.87%) than in the mock group (1.56% ± 0.96%, *p* < 0.05 for both), whereas TIC10 did not induce a significant increase in CD8+ T cells (2.30% ± 1.83%) (Figure 4C). There were no significant differences in the infiltration of CD4+ T cells, PD1+ T cells, B cells, or NK cells, between mice in the mock, TIC10, LD‐EM, and LD‐EM + chemotherapy groups (Figure 4C–F). In myeloid‐derived immune cells, the percentage of total macrophages did not differ significantly among the treatment groups (Figure 4G). Notably, the percentage of M1 macrophages was significantly higher in the LD‐EM group (12.73% ± 4.80%, *p* < 0.05) and combination therapy group (21.60% ± 1.47%, *p* < 0.001) than in the mock group (1.87% ± 0.73%), whereas TIC10 did not significantly increase the percentage of M1 macrophages (1.36% ± 0.55%) (Figure 4H). By contrast, the percentage of M2 macrophages was significantly lower in the LD‐EM group (4.17% ± 2.02%) and combination therapy group (3.05% ± 2.29%) than in the mock group (15.20% ± 2.46%, *p* < 0.01 for both), whereas TIC10 did not significantly decrease the percentage of M2 macrophages (13.05% ± 3.86%) (Figure 4H). Additionally, the percentage of dendritic cells (DCs) was significantly higher in the LD‐EM group (8.49% ± 4.38%) and combination therapy group (6.62% ± 1.72%) than in the mock group (1.90% ± 1.30%, *p* < 0.05 for both), whereas TIC10 significantly decreased the percentage of DCs (0.56% ± 0.29%, *p* < 0.05) (Figure 4I). Finally, the percentage of MDSCs was significantly lower in the LD‐EM group (6.00% ± 3.64%) and combination therapy group (6.01% ± 2.37%) than in the mock group (7.04% ± 2.91%, *p* < 0.05 for both), whereas TIC10 significantly increased the percentage of MDSCs (13.37% ± 1.94%, p < 0.05) (Figure 4J). Next, we analyzed the immune status of the TME through IHC analysis. The combination therapy group exhibited increased levels of CD3+ and CD8+ T cells, but not CD4+ T cells or CD4 + PD+ cells, which were consistent with the flow cytometry analysis (Figure S2). Additionally, IHC analysis of immune checkpoint markers PD‐1/PD‐L1, CTLA‐4, and LAG‐3 revealed no significant differences among the four groups (Figure S2).

**FIGURE 5:**
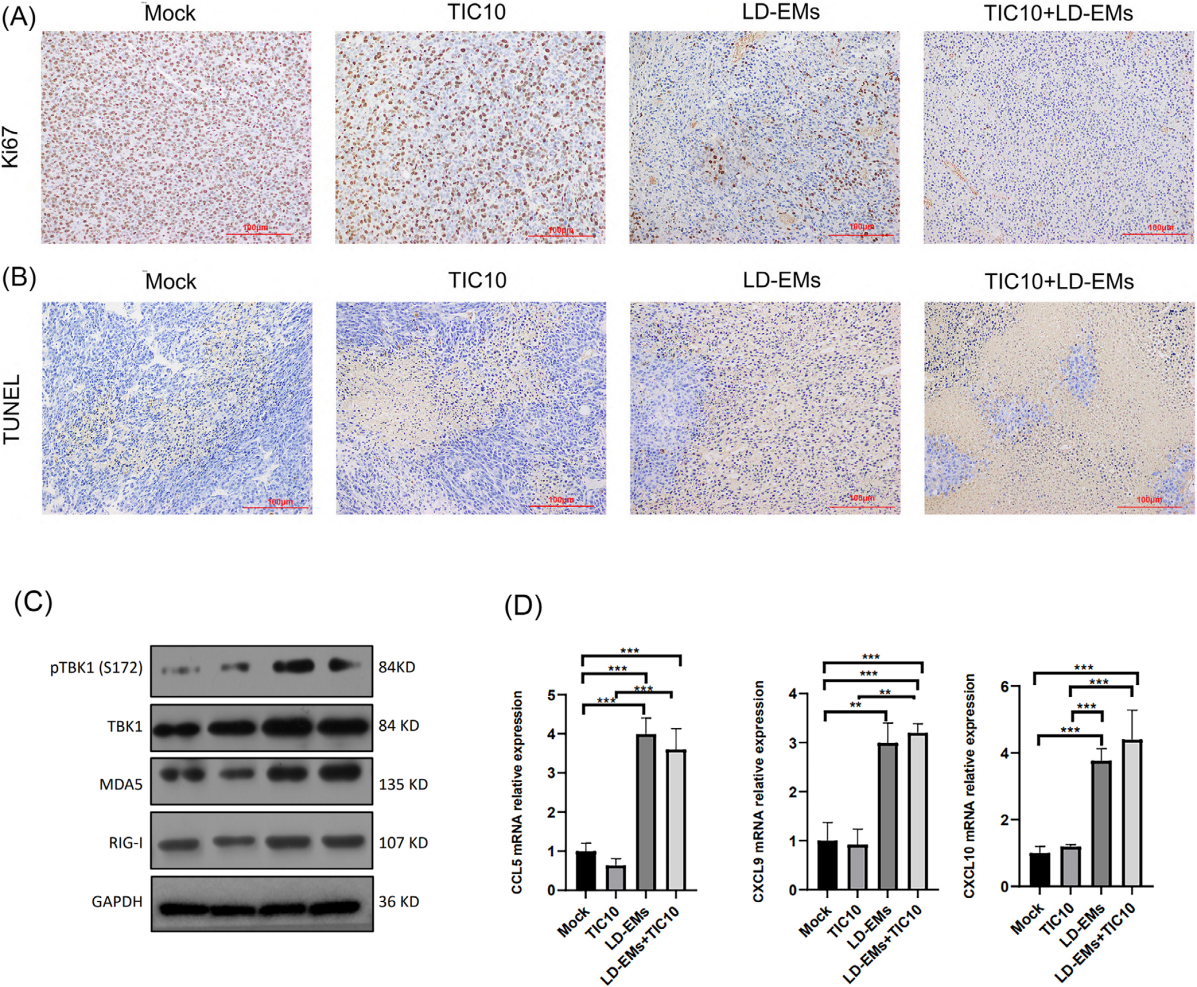
The possible mechanism of TIC10, LD‐EMs alone, or in combination on tumor in vivo. (A) IHC staining to analyze tumor proliferation by Ki67 staining; (B) Representative images of tumor necrosis by TUNEL staining. Slides were captured at 200× magnification. (C) The protein expression levels of ERV signaling were measured by western blot in tumors of different groups at the end of the in vivo experiment (D) The relative mRNA levels of chemokine genes were measured via qRT–PCR analysis.

Further analysis revealed that both LD‐EMs and combination therapy significantly decreased Ki67 expression, suggesting reduced tumor proliferation (Figure 5A). A concurrent increase in TUNEL staining was observed in the tumor sections, reflecting greater levels of tumor necrosis (Figure 5B). After LD‐EMs treatment, key viral RNA‐sensing proteins, including MDA5 and RIG‐I, were stimulated. We speculated that LD‐EMs treatment restored type I IFN signaling in CT26 mouse models, as indicated by the increase in TBK and TBK phosphorylation (Figure 5C). In addition, the transcription and secretion of the chemokines CCL5, CXCL9, and CXCL10 were promoted after treatment in LD‐EMs and the combination group (Figure 5D). The results indicated that LD‐EMs induce the interferon response and promote chemokine production by inducing viral mimicry and activating the ERV signaling pathway. Taken together, the antitumor effect of LD‐EMs was attributed, at least in part, to the induction of a viral mimicry response.

### 3.4 Combined Efficacy of LD‐EMs With TIC10 In Vitro

**FIGURE 6:**
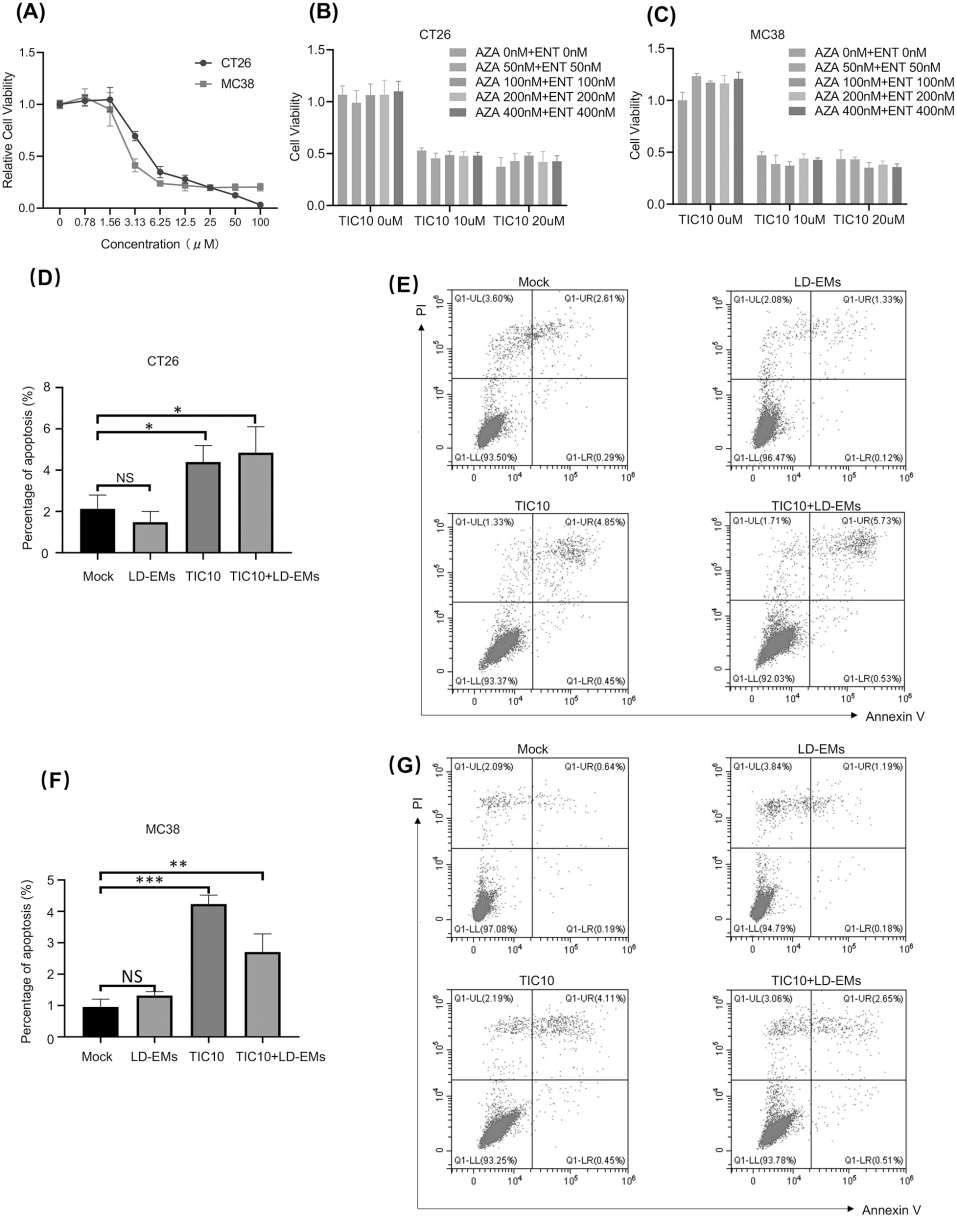
The effect of TIC10, LD‐EMs alone, or in combination on tumor cells in vitro. (A) CT26 and MC38 cells were treated with different concentrations (0–100 μM) of TIC10 for 48 h. The Cell Counting Kit‐8 (CCK8) assay was used to measure optical density at 450 nm, and cell viability was calculated according to the manufacturer's guidelines. (B) CT26 cells were treated with specific concentrations of 5‐azacitidine and (or) entinostat and (or) TIC10, and then incubated for 48 h. Subsequently, the CellTiter‐Lumi Luminescence Detection Reagent was introduced to detect chemiluminescence signals. (C) MC38 cells were treated with specific concentrations of 5‐azacitidine and (or) entinostat and (or) TIC10, and then incubated for 48 h. Subsequently, the CellTiter‐Lumi Luminescence Detection Reagent was introduced to detect chemiluminescence signals. (D) CT26 cells were treated with 10 μM TIC10 and (or) LD‐EMs (100 nM 5‐Aza, 100 nM entinostat) for 48 h and stained with Annexin V‐FITC/7‐AAD. (E) Representive images of CT26 cell apoptosis. Sums of percentages of early apoptosis (Q1‐LR) and late apoptosis (Q1‐UR) were calculated as total apoptosis ratios. (F) MC38 cells were treated with 10 μM TIC10 and (or) LD‐EMs (100 nM 5‐Aza, 100 nM entinostat) for 48 h and stained with Annexin V‐FITC/7‐AAD. (G) Representive images of MC38 cell apoptosis. Sums of percentages of early apoptosis (Q1‐LR) and late apoptosis (Q1‐UR) were calculated as total apoptosis ratios. Results are representative of three independent experiments. Data were analyzed by flow cytometry. *N* = 3. All bars represent mean ± SD. *p < 0.05; **p < 0.01; ***p < 0.001.

To further illustrate the mechanism underlying the synergistic efficacy of LD‐EMs and TIC10, we explored their individual and combined effects on proliferation and apoptosis. First, we conducted a dose–response assessment of TIC10 in CT26 and MC38 cells, which revealed a gradual decrease in cell viability with increasing concentrations. The calculated half‐maximal inhibitory concentrations for MC38 and CT26 were 10.55 and 10.14 μM, respectively (Figure 6A). We selected a TIC10 concentration of 10 μM for subsequent analyses. Cell viability analysis was performed using CellTiter‐Lumi at various concentrations of TIC10 and EMs (5‐Aza and entinostat) (Figure 6B,C). Notably, the EMs (5‐Aza and entinostat) did not suppress cell viability, even at a concentration of 400 nM (*p* > 0.05). A concentration of 100 nM for 5‐Aza and entinostat was then applied in subsequent analyses, which had exerted a significant inhibitory effect on MDSCs in our previous study [13]. The effect of TIC10 in combination with LD‐EMs on induction of tumor cell death was further evaluated through flow cytometry analysis. The tumor cell lines CT26 and MC38 were treated with vehicle control, single‐agent TIC10, LD‐EMs (5‐Aza and entinostat), and the combination of TIC10 and LD‐EMs for 48 h (Figure 6D–G). In the CT26 cell line, the rate of cell apoptosis was significantly higher when the cells were treated with TIC10 (4.40% ± 0.79%) and the combination of TIC10 and LD‐EMs (4.85% ± 1.26%) than in the mock group (2.13% ± 0.67%, *p* < 0.05 for both), whereas treatment with LD‐EMs alone did not induce a significant increase in apoptosis (1.48% ± 0.52%, *p* > 0.05) (Figure 6D,E). Similar results were observed in the MC38 cell line, in which cell apoptosis significantly increased upon treatment with TIC10 (4.24% ± 0.28%, *p* < 0.001) or the combination of TIC10 and LD‐EMs (2.71% ± 0.58%, *p* < 0.01) compared with the mock group (0.96% ± 0.25%), whereas treatment with LD‐EMs alone did not significantly increase apoptosis (1.32% ± 1.13%, *p* > 0.05) (Figure 6F,G). However, the combination group did not show more apoptosis than single‐agent treatment with TIC10, indicating that LD‐EMs did not show combinatorial efficacy with TIC10 in vitro, unlike the effect in vivo, where the TME may play an important role.

## 4 Discussion

This study demonstrated that the combination of LD‐EMs and TIC10 may be an efficacious strategy in patients with GI cancer. The success of this combination therapy acted both on the tumor cells and within the TME. Although TIC10 directly induces tumor cell apoptosis, LD‐EMs modulate the immune microenvironment by increasing the percentages of CD45+ lymphocytes, CD3+ T cells, M1 macrophages, and dendritic cells while reducing the percentages of M2 macrophages and MDSCs, suggesting the complementary and synergistic effects of TIC10 and LD‐EMs.

The observed shifts in immune cell subpopulations under treatment with LD‐EMs and combination therapy suggest a reprogramming of the TME toward a more immunostimulatory state. The increased presence of CD45+ lymphocytes—including T cells, B cells, and NK cells—suggests enhanced immune infiltration into the TME [26]. The rise in CD3+ T cells highlights an adaptive immune response, likely driven by improved antigen presentation from dendritic cells and modulation of the cytokine milieu, fostering T‐cell activation [27]. M1 macrophages, known for their pro‐inflammatory and anti‐tumorigenic functions, exhibit an increased proportion, suggesting a shift in macrophage polarization toward an inflammatory phenotype. This change is likely mediated by cytokines such as interferon‐gamma (IFN‐γ) and granulocyte‐macrophage colony‐stimulating factor (GM‐CSF), which drive M1 differentiation and function [28]. Conversely, the reduction in M2 macrophages [29] and MDSCs [30] alleviates immunosuppressive mechanisms that promote tumor progression. The increased proportion of DCs, professional antigen‐presenting cells, implies enhanced antigen processing and presentation, leading to more efficient T‐cell priming and activation [31]. In contrast, myeloid‐derived suppressor cells (MDSCs), potent inhibitors of T‐cell activity that contribute to immune evasion within the TME, are decreased, indicating reduced myeloid cell‐mediated immunosuppression [30]. Overall, the combination therapy appears to enhance tumor antigen presentation, promote effector immune cell infiltration, and suppress immunosuppressive mechanisms, thereby fostering a more effective antitumor immune response.

TIC10, initially identified as an activator of cell demise by increasing the levels of TRAIL ligand and its surface receptor DR5 [32], is presently undergoing clinical evaluation in patients with diverse solid tumors. In addition to inducing caspase‐dependent apoptosis, TIC10 also suppresses Akt and ERK signaling, which normally promote tumor cell survival, thereby enhancing tumor sensitivity to apoptosis [33, 34]. Conventional chemotherapy not only kills tumor cells directly but also engages the immune system to enhance antitumor effects. Understanding these immune‐based mechanisms is crucial for optimizing chemo‐immunotherapy combinations to improve cancer treatment outcomes [35, 36]. Similarly, beyond its direct cytotoxic effects, TIC10 modulates the immune microenvironment by influencing cytokine secretion, which may indirectly alter myeloid cell populations [37]. Notably, our study found that TIC10 treatment induced a notable reduction in DCs and a corresponding increase in MDSCs, which may offer insight into the unsatisfactory antitumor outcomes. One possible explanation is that TIC10‐induced TRAIL activates the noncanonical NFκB2 pathway in cancer cells, leading to the production of cytokines such as IL‐6, IL‐10, and GM‐CSF [38]. These cytokines promote the expansion and recruitment of myeloid‐derived suppressor cells (MDSCs) [39], which in turn inhibit dendritic cell (DC) differentiation. Additionally, cytokines in myeloid progenitors can skew differentiation toward MDSCs instead of DCs [40]. Moreover, once expanded, MDSCs can suppress DCs through mechanisms such as arginase activity, reactive oxygen species, and nitric oxide (NO) production [41]. However, the exact mechanisms remain to be fully elucidated. Although the direct impact of TIC10 on MDSCs remains less thoroughly explored, its broader effects on the TME may contribute to MDSCs recruitment and functional suppression of immune responses.

Given its distinctive mode of action and impact on the TME, TIC10 has emerged as a promising candidate for synergistic therapies aimed at overcoming immune suppression. Combination therapy has been attempted to sensitize the effectiveness of TIC10, and epigenetic therapies have been explored. Early preclinical research has shown that histone deacetylase (HDAC) inhibitors can activate the TNF superfamily member 10 promoter, thereby increasing TRAIL expression [42]. The combination of HDAC inhibitors and TRAIL agonists can restore the sensitivity in resistant cancers toward TRAIL‐induced apoptosis [43, 44]. Furthermore, investigations have revealed the potential synergy between TIC10 and EMs such as EZH2 inhibitors and HDAC inhibitors across multiple cancer types, substantiating the rationale for such combination strategies [45]. LD‐EMs such as 5‐Azacytidine and histone deacetylase (HDAC) inhibitors can reprogram immune cells, promoting an antitumor immune response by increasing MHC class I/II expression and antigen presentation, thereby enhancing T cell activation and infiltration. Additionally, they can suppress STAT6 signaling, which is associated with M2 macrophage polarization, thereby shifting macrophage balance toward the pro‐inflammatory M1 phenotype. LD‐EMs also activate NF‐κB and IFN‐γ pathway, which further enhance immune responses. IFN‐γ signaling supports M1 macrophage polarization, boosting tumoricidal activity, whereas NF‐κB activation in dendritic cells improves antigen presentation and cross‐priming of T cells, leading to a more effective adaptive immune response. Additionally, LD‐EMs have been shown to reduce the viability of granulocytic MDSCs, possibly by downregulating Rb‐dependent pathways involved in myeloid differentiation [46]. Regarding tumor cells, LD‐EMs have minimal impact. In our previous study, we found that low‐dose 5‐Aza and entinostat had minimal impact on the proliferation, viability, and apoptosis of tumor cells in vitro. Similarly, in a corresponding in vivo experiment, low‐dose 5‐Aza (0.5 mg/kg/day) and entinostat (5 mg/kg/day) failed to slow tumor growth in NSG mouse models, suggesting a restricted effect on tumor cells [13]. The current study corroborates these findings, demonstrating that LD‐EMs exerted no substantial effect on cell viability or tumor cell apoptosis in vitro, which is similar to our previous study. In summary, by combining TIC10's tumoricidal activity with LD‐EMs' ability to enhance immune function, this therapeutic strategy may create a positive feedback loop in which tumor cell death promotes antigen release, further stimulating DCs activation and T‐cell priming.

Nonetheless, the specific interactions between TIC10 and the TME need further investigation, particularly regarding the mechanisms underlying the effects of TIC10 and combination therapy on the dynamics and function of MDSC and DC populations. Given the immunosuppressive effects associated with TIC10, future studies should explore additional combination strategies, such as immune checkpoint inhibitors or MDSC‐targeting agents, to counteract these effects. Furthermore, the validation of these findings in a broader spectrum of preclinical models is imperative to establish their robustness and translational potential.

## 5 Conclusions

In summary, our investigation highlights the distinct yet complementary mechanisms of TIC10 and LD‐EMs. A combined regimen involving LD‐EMs and TIC10 represents a rational and viable therapeutic approach for patients with advanced and metastatic GI cancers by simultaneously inducing tumor cell apoptosis and reprogramming the immune microenvironment to enhance antitumor immunity.

## Author Contributions

**Jianling Zou:** data curation (lead); formal analysis (equal); investigation (equal); validation (lead); visualization (equal); project administration (equal); writing – original draft (equal); writing – review and editing (equal); funding acquisition (equal). **Wentao Yang:** data curation (supporting); formal analysis (supporting); methodology (supporting); visualization (equal). **Shuang Li:** data curation (supporting); validation (equal); resources (equal); funding acquisition (supporting); writing – review and editing (equal). **Fei Liu:** data curation (supporting); resources (supporting); writing – review and editing (supporting); funding acquisition (supporting). **Jinjia Chang:** conceptualization (equal). **Wenhua Li:** conceptualization (supporting); methodology (equal); software (equal). **Mingzhu Huang:** conceptualization (equal); methodology (supporting); resources (supporting); software (equal); supervision (equal). **Xiaodong Zhu:** methodology (equal); resources (equal); supervision (supporting); **Jianyin Zou:** investigation (equal); writing – original draft (equal); writing – review and editing (supporting); methodology (supporting). **Weijian Guo:** formal analysis (equal); project administration (equal); writing – review and editing (supporting); **Zhiyu Chen:** project administration (lead); writing – review and editing (equal); supervision (equal); funding acquisition (equal).

## Ethics Statement

The studies involving animal studies were approved by the Ethics Committee of Fudan University Shanghai Cancer Center.

## Consent

The authors have nothing to report.

## Conflicts of Interest

The authors declare no conflicts of interest.

## Supporting information


**Figure S1.** Gating strategy used to identify the immune profile after treatment.

**Figure S2.** The immune status analysis by IHC after treatment with TIC10, LD‐EMs, or combination therapy.

**Table S1.** Flow cytometry reagent.

## Data Availability

All data generated or analyzed during this study are included in this published article (and its additional information files). Further inquiries can be directed to the corresponding author.
