# Supplementary figures and images for: Combination of LowDose Epigenetic Modifiers and TIC10 for the Activation of Antitumor Immunity and Inhibition of Tumor Growth in Gastrointestinal Cancer
Source: Cancer Med. 2025 Jul 17;14(14):e71061. doi: 10.1002/cam4.71061 (PMC12268316; doi:10.1002/cam4.71061)

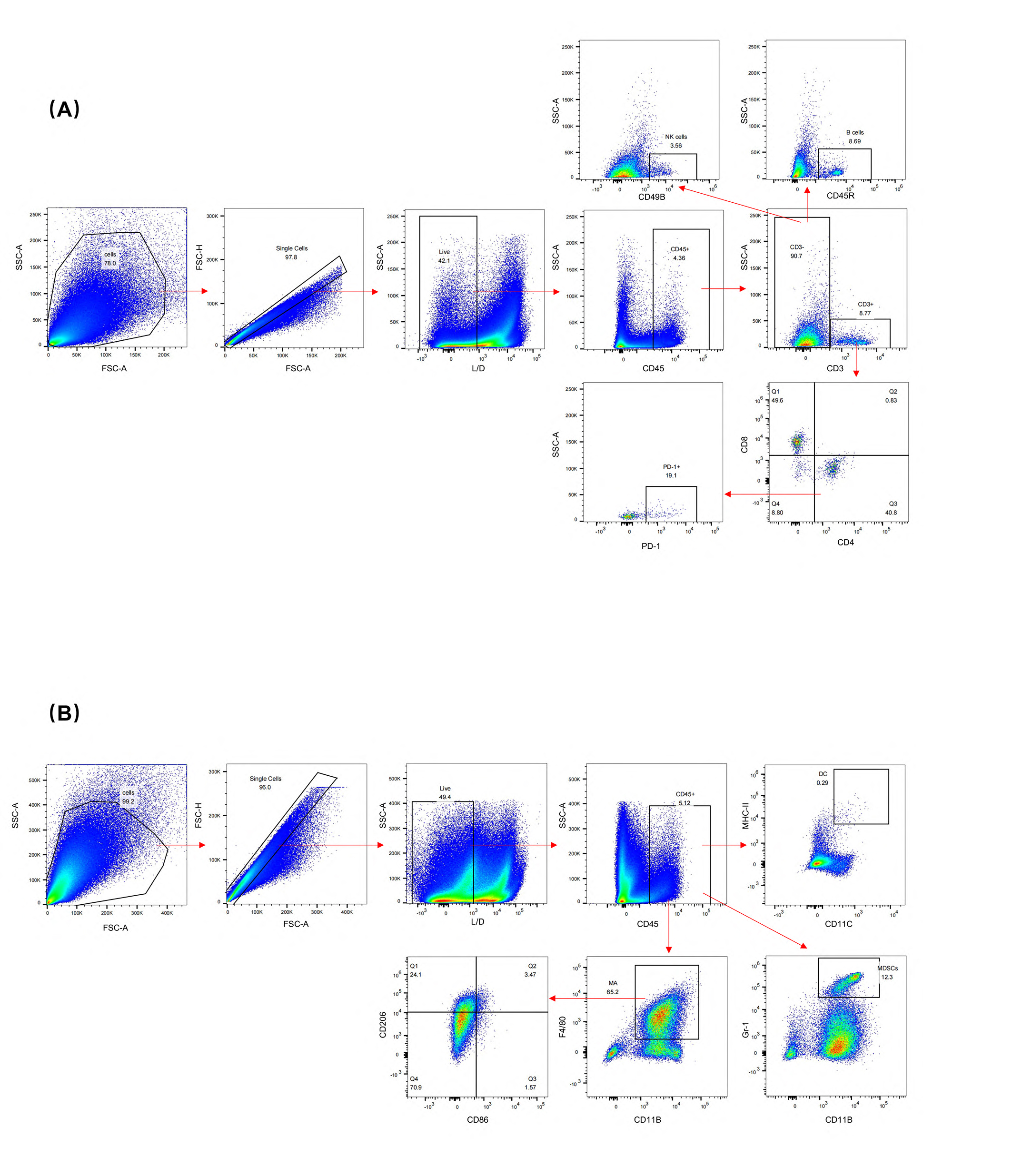

Supplement: Supplementary file 1 — Figure S1. Gating strategy used to identify the immune profile after treatment. [file CAM4-14-e71061-s003.jpg]

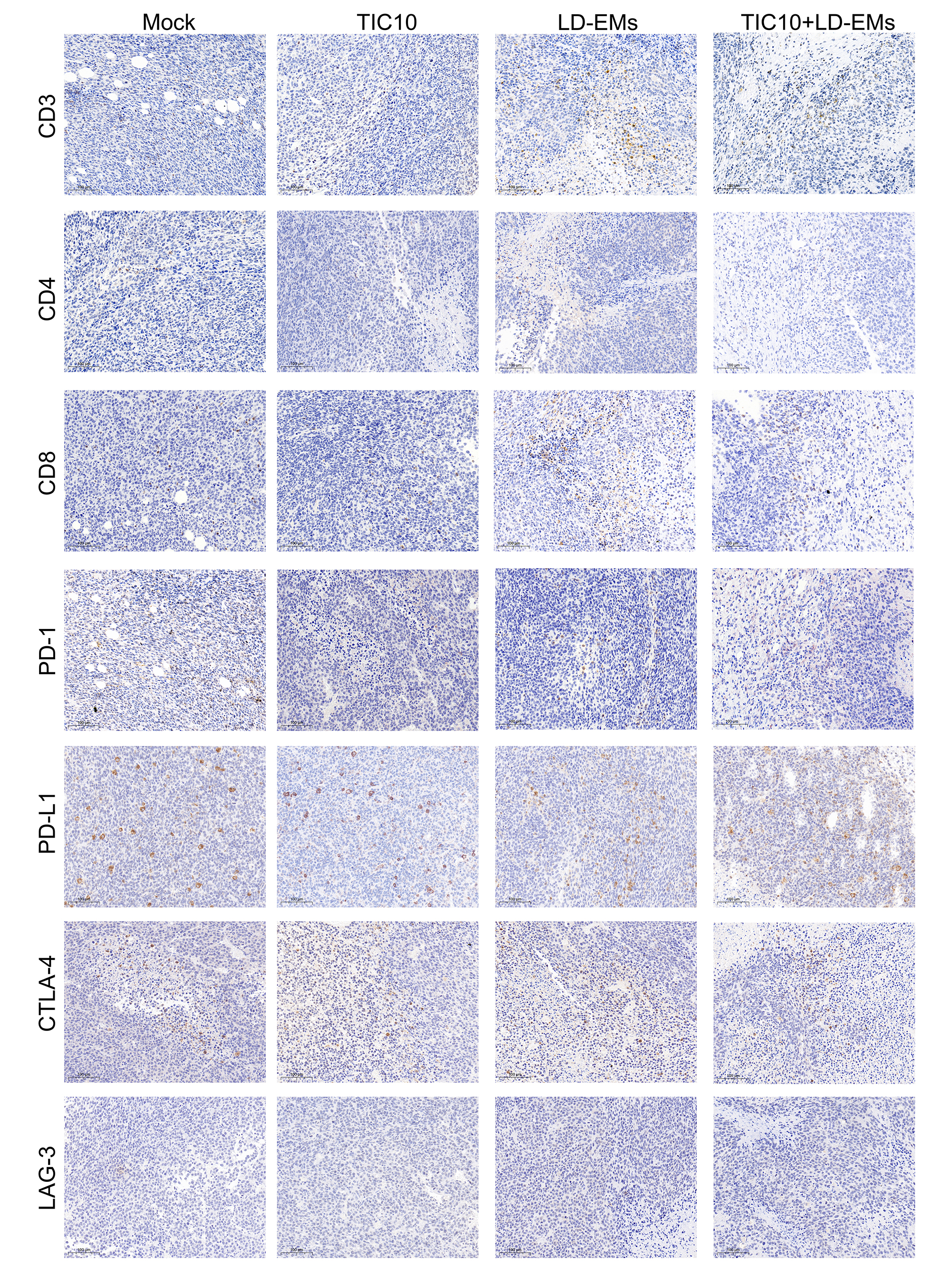

Supplement: Supplementary file 2 — Figure S2. The immune status analysis by IHC after treatment with TIC10, LD‐EMs, or combination therapy. [file CAM4-14-e71061-s001.jpg]
