# Supplementary material for: Combination of LowDose Epigenetic Modifiers and TIC10 for the Activation of Antitumor Immunity and Inhibition of Tumor Growth in Gastrointestinal Cancer
Source: Cancer Med. 2025 Jul 17;14(14):e71061. doi: 10.1002/cam4.71061 (PMC12268316; doi:10.1002/cam4.71061)
Supplement: Supplementary file 3 — Table S1. Flow cytometry reagent. [file CAM4-14-e71061-s002.docx]

Supplementary Table 1

## Table S1: Flow Cytometry Reagent

| Flow Cytometry Reagent | SOURCE | Catalog# |
| --- | --- | --- |
| APC-Cy7 Rat Anti-Mouse CD45(30-F11) | BD Pharmingen | 557659 |
| BB700 Armenian Hamster Anti-Mouse CD3e(145-2C11) | BD Pharmingen | 566494 |
| FITC Rat Anti-Mouse CD4(RM4-5) | BD Pharmingen | 553046 |
| Alexa Fluor 647 Rat Anti-Mouse CD8a(53-6.7) | BD Pharmingen | 557682 |
| PE Hamster Anti-Mouse CD49b(HMα2) | BD Pharmingen | 558759 |
| BV510 Rat Anti-Mouse B220/CD45R(RA3-6B2) | BD Pharmingen | 563103 |
| BV421 Hamster Anti-Mouse CD279 (PD-1)(J43) | BD Pharmingen | 562584 |
| Fixable Viability Stain 700 | BD Pharmingen | 564997 |
| FITC Rat Anti-CD11b(M1/70) | BD Pharmingen | 557396 |
| BV421 Rat Anti-Mouse F4/80(T45-2342) | BD Pharmingen | 565411 |
| BV786 Rat Anti-Mouse CD86(GL1) | BD Pharmingen | 740877 |
| Alexa Fluor 647 Rat Anti-Mouse CD206(MR5D3) | BD Pharmingen | 565250 |
| PE-Cy7 Hamster Anti-Mouse CD11c(HL3) | BD Pharmingen | 558079 |
| BV605 Rat Anti-Mouse I-A/I-E(M5/114.15.2 ) | BD Pharmingen | 563413 |
| PE Rat Anti-Mouse Ly-6G and Ly-6C(RB6-8C5) | BD Pharmingen | 553128 |
| Stain Buffer (BSA) | BD Pharmingen | 554657 |
| Fixation/Permeablization Kit | BD Pharmingen | 554714 |
| Purified Rat Anti-Mouse CD16/CD32 (Mouse BD Fc Block)(2.4G2) | BD Pharmingen | 553142 |
| Tumor Dissociation Kit, mouse | Miltenyi | 130-096-730 |
| FITC Annexin V Apoptosis Detection Kit I | BD Pharmingen | 556547 |

**Supplementary Figure legends**

**Figure S1.** Gating strategy used to identify the immune profile after treatment.

(A) Gating strategy used to identify and analyze CD45+ cells, B cells, NK cells, T cells, and PD1+CD4+ T cells by FACS. (B) Gating strategy used to identify and analyze myeloid cells, including M1 and M2 macrophages, dendritic cells, and MDSCs by FACS.

**Figure S2.** **The immune status analysis by IHC after treatment with TIC10, LD-EMs, or combination therapy.**

Representative images of IHC staining to analyze T cell infiltration by CD3, CD4 and CD8 staining, the immune checkpoints of PD-1/PD-L1, CTLA-4 and LAG-3. Slides were captured at 200x magnification.
